# Drunk or Dying

**Published:** 1898-01-29

**Authors:** 


### EDITOR'S LETTER-BOX.

[Our Correspondents are reminded that prolixity is a great bar to publication, and that brevity of style and conciseness of statement greatly facilitate early insertion.]

### "DRUNK OR DYING."

Mr. Leonard D. Rea, Secretary of the West Ham Hospital, writes: In reference to your "Annotation" in this week's Hospital, headed "Drunk or Dying?" may I call your attention to the accompanying newspaper paragraph? In the letter therein referred to, the only question dealt with is the allegation that the patient was unconscious while at the hospital, as on this point alone the censure of the jury is based. I think nearly every unprejudiced person will agree with the views which you so clearly express, and, in justice to the hospital which I have the honour to represent, I should mention that we have a small room set apart for the reception of troublesome cases which might prove hurtful to other patients. On the night on which this particular patient was treated our accommodation in this respect was severely taxed, there being already in hospital three cases suffering from alcoholic excess and slight head injuries. It is exactly in this category that the diagnosis of the house surgeon (after careful examination) placed the deceased, and it is one of the ironies of life that the three patients detained should grow decently sober—no serious injury being revealed—and that the one sent away should die. I am thoroughly convinced that the house surgeon—against his own interest as the sequel proved—acted solely with a conscientious regard to the interests of the charity—that is, of the suffering poor by whom its existence is justified. If in your judgment the circumstances I mention should be made public, I will with confidence leave the matter in your hands, appreciating the spirit of fair play which actuates the conduct of your valuable journal.

*** The following is the letter referred to:—

"West Ham Hospital, Stratford, E., Jan. 13, 1898.

"To Dr. Alexander Ambrose, Coroner.—Sir,—Your letter of the 8th inst. was laid before the committee of management of this hospital at their monthly meeting, 18 members being present, including the medical board. The committee, after calling before them Dr. Macdonald and the sister who was in attendance when the late Mr. A. S. Woolmer was treated at the hospital, and fully investigating the facts and the treatment that the patient received, unanimously carried a resolution exonerating him from all blame in regard to it. It may be added that the members of the committee were satisfied from the evidence they had before them that the deceased was not unconscious during the time he was in the hospital, nor when he left it; that he was not carried out, and that he spoke. Will you kindly make this communication known to the foreman of the jury?—I am, sir, your obedient servant, Leonard D. Rea, Secretary."

We cannot but feel that it was a pity that the facts mentioned in Mr. Rea's letter were not brought to the notice of the coroner at the time of the inquest. Many a scandal would be nipped in the bud if hospitals were properly represented at inquests.—Ed. *T. H.*

